# Depigmentation Effect of Kadsuralignan F on Melan-A Murine Melanocytes and Human Skin Equivalents

**DOI:** 10.3390/ijms14011655

**Published:** 2013-01-15

**Authors:** Myeong-Jin Goh, Hae-Kwang Lee, Liang Cheng, De-Yun Kong, Jae-Ho Yeon, Quan-Quan He, Jun-Cheol Cho, Yong Joo Na

**Affiliations:** 1 Skin Research Institute, Amorepacific R&D Center, 314-1 Bora-dong, Giheung-gu, Yongin 446-729, Korea; E-Mails: stcanopus@amorepacific.com (M.-J.G.); haekwang@amorepacific.com (H.-K.L.); jccho@amorepacific.com (J.C.); 2 Department of Traditional Chinese Medicine, Shanghai Institute of Pharmaceutical Industry, 1320 Beijing Road (W), Shanghai 200040, China; E-Mails: liangcheng0406@hotmail.com (L.C.); deyunk@yahoo.com.cn (D.-Y.K.); 3 Amorepacific Shanghai R&I Center, 383 Yumin Road, Jiading District, Shanghai 201801, China; E-Mails: jhyeon@amorepacific.com (J.-H.Y.); hequanquan@cn.amorepacific.com (Q.-Q.H.)

**Keywords:** kadsuralignan F, melanogenesis, tyrosinase degradation

## Abstract

The development of melanogenic inhibitors is important for the prevention of hyperpigmentation, and, recently, consideration has been given to natural materials or traditionally used ingredients such as Chinese medicine. The aim of this study is the evaluation of a new anti-melanogenic candidate, kadsuralignan F, from the natural plant *Kadsura coccinea*, as well as the determination of mechanisms of melanogenesis inhibition at a molecular level. Kadsuralignan F significantly reduced melanin synthesis in a dose-dependent manner in a murine melanocyte cell line and human skin equivalents. There was no direct inhibition on mushroom tyrosinase or cell-extract tyrosinase activity, and mRNA expression of tyrosinase and other melanogenic genes such as tyrosinase-related protein-1 (trp-1) or trp-2 were not affected by kadsuralignan F. Interestingly, the protein level of tyrosinase was dramatically downregulated with kadsuralignan F treatment. We found that a decrease of tyrosinase protein by kadsuralignan F was fully recovered by MG132, a proteasome inhibitor, but not by chloroquine, a lysosome inhibitor. In this study, we found that kadsuralignan F, a lignan from an extract of *Kadsura coccinea*, has an inhibitory activity on melanin synthesis through tyrosinase degradation. These findings suggest that kadsuralignan F can be used as an active ingredient for hyperpigmentation treatment.

## 1. Introduction

Melanin synthesis and distribution contributes to mammalian skin color [1]. Melanin pigments have a role in the protection of skin from ultraviolet irradiation, as well as various oxidative stresses [2]. However, irregular synthesis of melanin may cause problems with the skin, and there are a number of hyperpigmentary disorders or conditions attributed to this, such as melasma, solar lentigo, post-inflammatory hyperpigmentation, or freckles [3]. Thus, effective skin whitening ingredients could be useful for clinical therapy, as well as for cosmetic applications.

Melanin is synthesized in the melanosome, a unique organelle in melanocytes [4]. There are three types of melanogenic enzymes in the melanosome: tyrosinase, tyrosinase-related protein (TRP)-1 and TRP-2. It is well known that tyrosinase, a type I membrane glycoprotein, plays the most critical role in melanin synthesis [5]. Tyrosinase participates in the critical rate-limiting step in melanin synthesis. Tyrosinase catalyzes the two initial steps in melanogenesis, the hydroxylation of tyrosine to dihydroxyphenylalanine (DOPA) and the oxidation of DOPA to DOPA-quinone [6]. Tyrosinase is synthesized in the endoplasmic reticulum (ER), and is processed by post-translational modifications, including *N*-glycosylation in the Golgi apparatus [7]. After maturation in the Golgi, tyrosinase moves through the trans-Golgi network to melanosomes for melanin synthesis, or to the degradation machinery as a regulatory process for balance between synthesis and degradation of the protein [8]. Tyrosinase is degraded by two pathways; the proteolysis ER associated protein degradation (ERAD) in the ubiquitin proteasome system (UPS) [9], or the endosomal/lysosomal degradation system [10]. Because of the key regulatory role of tyrosinase on melanogenesis, many previous studies have focused on tyrosinase inhibitors, especially those occurring from natural sources. The stimulation of tyrosinase degradation has been become a new target in discovering skin-whitening agents [8,11]. Substances such as phenylthiourea [10], phospholipase D2 [12], inulavosin [13], terrein [14], and dimethoxytolyl propylresorcinol [15] can induce tyrosinase degradation, resulting in reduced melanin synthesis.

*Kadsura coccinea* (Lem.) A. C. Smith (Schizandraceae) is widely distributed throughout southwest mainland China. The dried roots of *K. coccinea*, called *Hei Lao Hu* in Chinese, are used as a traditional medicine for the treatment of gastric and duodenal ulcers, chronic gastritis, acute gastroenteritis, and rheumatoid arthritis [16]. Previous phytochemical and biological investigations of *K. coccinea* have yielded some lignans and triterpenoids, and have identified their anti-tumor [17], nitric oxide inhibitory [18], and anti-HIV actions [19], as well as their protection of the liver [20]. Recently, Shu *et al.* reported the isolation of new lignans from the air-dried roots of *K. coccinea*, and elucidation of the structures and physicochemical properties [21]. Among them, a dibenzocyclooctadiene lignan showed high inhibition activity on melanin synthesis, and the IR and NMR spectra analysis revealed that the compound was identified as kadsuralignan F. In this study, we report the anti-pigmentation property of kadsuralignan F on melanogenesis, due to tyrosinase degradation. To our knowledge, it is the first molecular mechanism study on the anti-melanogenesis effects of kadsuralignan F.

## 2. Results and Discussion

**Figure 1:**
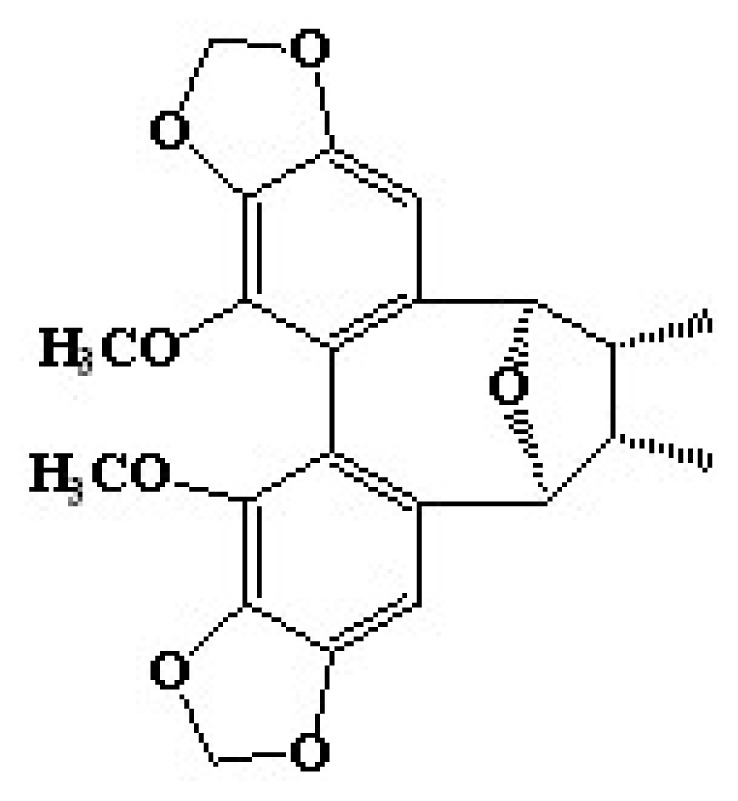
Chemical structure of kadsuralignan F.

Lignans are one of the main groups of phytoestrogens [22]. It is well known that phytoestrogens have physiological effects, such as estrogen-receptor binding and anti-oxidant activity effects [23,24]. Previous reports have shown various effects of lignans on skin: macelignan inhibited PAR-2 melanosome transfer in keratinocytes [25]; licarin E regulated MMP-1; and procollagen I was expressed in fibroblasts [26]. Kadsuralignans were isolated from *K. coccinea*, and their biological effects were reported, such as nitric oxide inhibition [18] and protection on t-butyl hydroperoxide-induced primary rat hepatocyte injury [27]. In this study, we first identify the anti-melanogenic effect, followed by the molecular mechanism of kadsuralignan F (Figure 1), a kadsuralignan.

**Figure 2:**
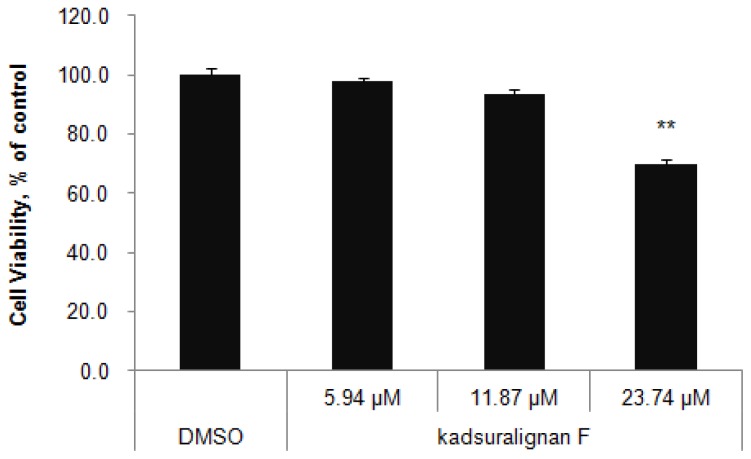
Effects of kadsuralignan F on the proliferation of melan-A cells. The cells were cultured with the indicated concentrations of kadsuralignan F for 3 days. Results are expressed as a percentage of the control (DMSO), and values are the average ± SE (standard error) of three determinations. **p < 0.01 *vs.* DMSO.

We used murine a melanocyte cell line melan-A cells to study melanogenesis inhibition by kadsuralignan F. Melan-A cells were cultured for three days at the indicated concentrations of kadsuralignan F, and cell viability was assessed by WST-1 assay (Figure 2). No significant change of cell viability was found in cells which were treated with up to 11.87 μM (5 ppm) kadsuralignan F, when compared with control cells (98% at 2.37 μM (1 ppm) and 94% at 11.87 μM, respectively). Therefore, further experiments in this study were conducted using concentrations less than 11.87 μM kadsuralignan F.

**Figure 3:**
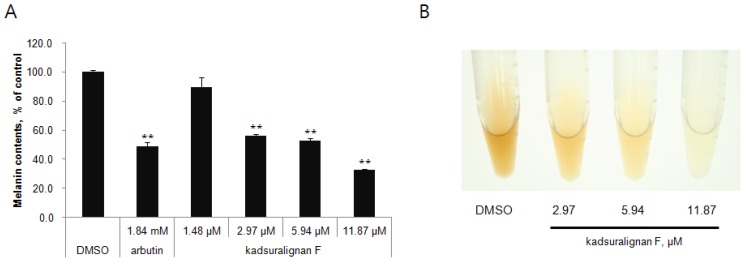
Effect of kadsuralignan F on melanin synthesis in melan-A cells. The melanin content of the cells was measured after 7 days of treatment with indicated concentrations of kadsuralignan F. (**A**) The melanin content was measured. Results are expressed as a percentage of the control (DMSO), and values are the average ± SE of three determinations. ** *p* < 0.01 *vs.* DMSO. (**B**) Photographs of melan-A cell pellet lysates. Cells were cultured with kadsuralignan F in 75 cm^2^ cell culture flasks for three days, were detached using trypsin-EDTA, and subsequently collected by centrifuge. Cell pellets were then dissolved in 2N NaOH containing 10% DMSO.

Quantification of melanin content indicated that kadsuralignan F significantly reduced the melanin synthesis in a dose-dependent manner (56% at 2.97 μM, 53% at 5.94 μM, and 33% at 11.87 μM) (Figure 3A). As shown in Figure 3B, the color of representative cell pellets clearly showed inhibitory activity of kadsuralignan F on melanogenesis, which became brighter with increasing concentration.

**Figure 4:**
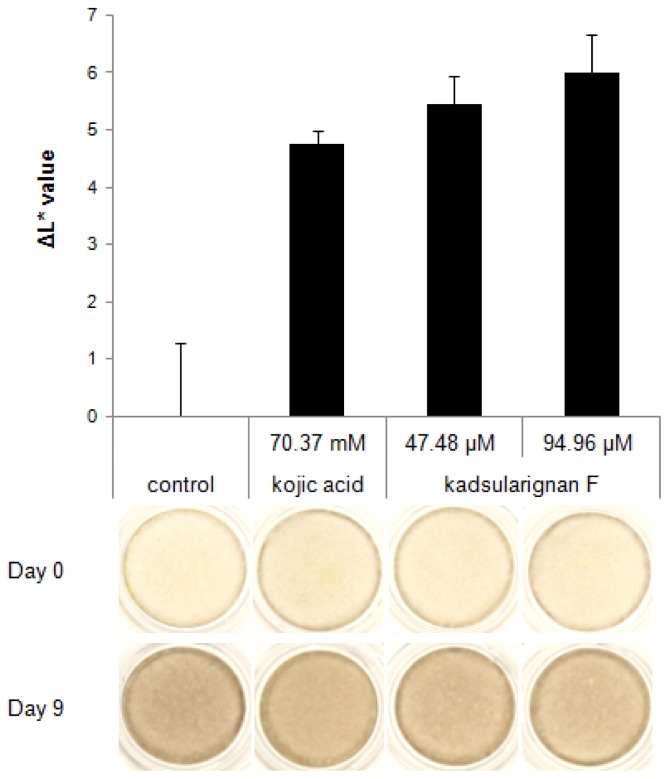
The effect of kadsuralignan F on human skin equivalents. Dark (from African-American skin, *n* = 2) MelanoDerms were treated with kadsuralignan F and kojic acid for nine days. Images were taken and the ΔL* value was calculated by comparing the increased L* values of each skin equivalent against a control.

The skin-whitening ability of kadsuralignan F was also tested in human skin equivalents. After nine days of treatment, pigmentation of kadsuralignan F-treated skin equivalents was compared against controls which were treated with the same volume of DPBS. Kadsuralignan F treatment at 47.48 μM and 94.96 μM (20 ppm and 40 ppm, respectively), which showed no cytotoxicity on skin equivalents, increased the brightness of the tissues relative to the control (Figure 4). Human skin equivalents, such as MelanoDerm, can implement many biological reactions that appear in physiological human skin, including interactions between melanocytes and keratinocytes, and skin pigmentation. Therefore, *in vitro* biosystems such as these are widely applied in many studies to evaluate the whitening efficacies of cosmetic and pharmaceutical agents. The result demonstrated that kadsuralignan F would be more effective for skin whitening than kojic acid, a proven whitening agent used in the test as a positive control [28], as the ΔL* value of the skin equivalents treated with 47.48 μM of kadsuralignan F was equal to that treated with 1% kojic acid (70.37 mM).

**Figure 5:**
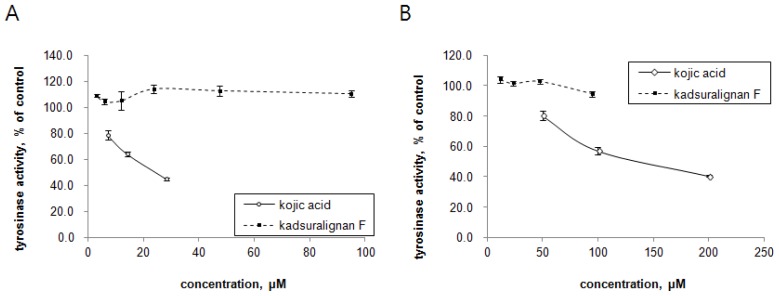
Effect of kadsuralignan F on tyrosinase activity *in vitro*. The inhibition effect on tyrosinase activity was evaluated using (**A**) mushroom tyrosinase and (**B**) melan-A cell extract by spectrophotometric methods. (A) Various concentrations of kadsuralignan F (■) and kojic acid (◇) were incubated with mushroom tyrosinase and tyrosine. (B) Various concentrations of kadsuralignan F (■) and kojic acid (◇) were incubated with melan-A cell extract and L-DOPA. Results are expressed as a percentage of the control, and values are the average ± SE of three determinations.

Since tyrosinase is the key regulatory enzyme for melanin production [5], the ability of kadsuralignan F on tyrosinase activity inhibition was investigated with mushroom tyrosinase and DOPA oxidase activity of mammalian tyrosinase. Tyrosinases from mushroom and melan-A cell extract were treated with different concentrations of either kadsuralignan F or kojic acid, and were incubated in the presence of substrates. In this cell-free assay system, we could try higher concentrations of kadsuralignan F compared to the cellular melanin assay, as it is more suitable for testing the direct inhibition of tyrosinase activity. As shown in Figure 5, kadsuralignan F had no significant inhibitory effect on tyrosine hydroxylase activity (Figure 5A) or DOPA oxidase activity (Figure 5B). Kojic acid, a well known tyrosinase inhibitor, suppressed both tyrosinase activities dose-dependently.

**Figure 6:**
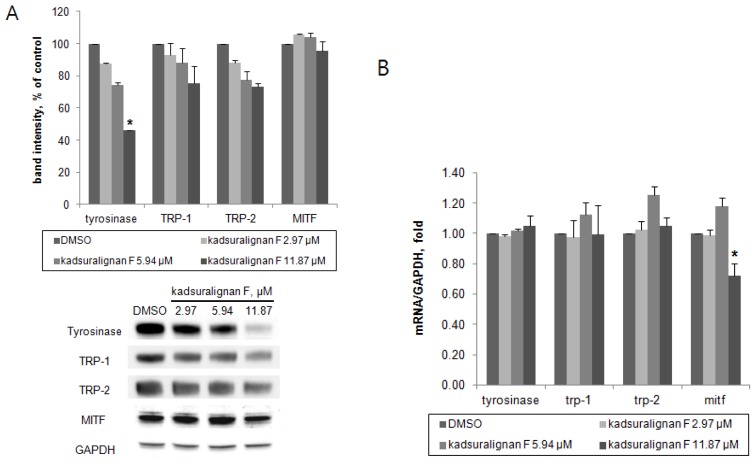
Effect of kadsuralignan F on melanogenesis-related gene expression of protein level and mRNA level in melan-A cells. (**A**) The cells were treated with the indicated concentrations of kadsuralignan F for three days, and Western blotting of tyrosinase, TRP-1, TRP-2, and MITF was performed. The loading control was assessed using anti-GAPDH antibody. The band intensities of melanogenic proteins were normalized by the band intensities of GAPDH as an internal control for each condition, and values are the average ± SE of three determinations. *p < 0.05 *vs.* DMSO; (**B**) The cells were cultured with kadsuralignan F for 24 h. mRNA levels were analyzed by real-time quantitative PCR. Gapdh was used as an internal standard, and melanogenesis-related gene mRNA/gapdh mRNA ratios are expressed relative to the control (DMSO), where values are the average ± SE of three determinations. *p < 0.05 *vs.* DMSO.

As there was no direct inhibition on tyrosinase activity by kadsuralignan F, further investigation of the expression of melanogenic proteins was performed. To investigate whether kadsuralignan F inhibits gene expression of melanogenic proteins, real-time reverse transcription polymerase chain reaction (qRT-PCR) assays were conducted in melan-A after 24 h treatment of kadsuralignan F. mRNA transcription levels of tyrosinase, trp-1, and trp-2 mRNA were not affected by kadsuralignan F (Figure 6B). Mitf, a master transcriptional regulator of melanogenic proteins was decreased to 72% levels of the untreated control at the highest concentration of kadsuralignan F applied. We also examined shorter time (2 and 6 h) treatment of kadsuralignan F, there was no significant change in those melanogenic genes expression (data not shown). Western blot analysis revealed that kadsuralignan F remarkably decreased the protein expression of tyrosinase (Figure 6A). The expression levels of TRP-1, TRP-2, and MITF were also diminished, but the decreases were not significant. Although it has been observed that the reduction in levels of Mitf gene expression was very low. Therefore, the amount of the MITF protein changes were expected to be low impact, and it was confirmed as a result of the Western blot experiment. These results suggest that melanin synthesis inhibition by kadsuralignan F did not result from the decrease of gene expression of melanogenic proteins, but was affected by the decrease in the expression level of tyrosinase protein, which might be related to post-translational modification processes of tyrosinase in melanocytes.

**Figure 7:**
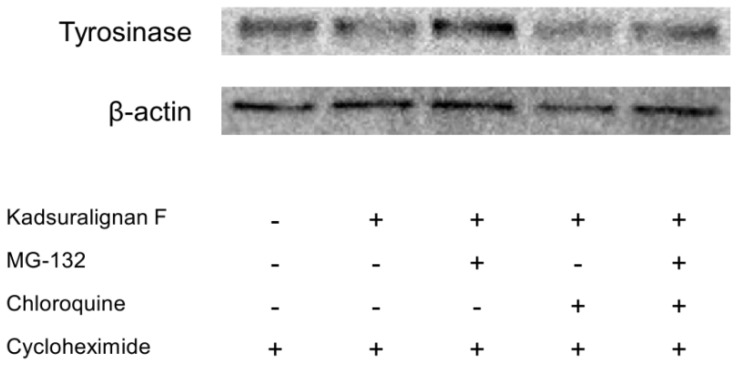
Effect of kadsuralignan F on tyrosinase degradation in melan-A cells. The cells were pretreated with cycloheximide, following treatment of proteolysis inhibitors (MG-132 and/or chloroquine), and they were then incubated with 11.87 μM kadsuralignan F. After treatment, Western blot analysis was conducted to analyze tyrosinase levels using whole cell lysates with anti-tyrosinase antibody. β-Actin was used as internal control for each condition.

Tyrosinase is endogenously removed by the ubiquitin-mediated proteasomal degradation system [8]. Recent studies have reported that agents degraded tyrosinase via activation of the UPS resulting in the acceleration of tyrosinase degradation [9,29]. To investigate whether tyrosinase degradation by kadsuralignan F is involved in proteasomal or lysosomal pathway, analysis using proteolysis inhibitors was conducted. Tyrosinase degradation is mediated via proteasome, and this degradation could affect melanin synthesis in melanocytes [30]. For an evaluation of kadsuralignan F on post-translational tyrosinase degradation, we introduced MG-132, a proteasome inhibitor, and/or chloroquine, a lysosomal proteolysis inhibitor. After 24 h of serum starvation, melan-A cells were treated with cycloheximide to inhibit protein synthesis, and then the proteolysis inhibitors were added for 1 h followed by 6 h of kadsuralignan F treatment. It was found that the tyrosinase decrease due to kadsuralignan F treatment was recovered by pretreatment with MG-132. However, chloroquine treatment did not show any effect on the tyrosinase recovery (Figure 7). These results indicate that proteasomal tyrosinase degradation was mediated by kadsuralignan F treatment. Further study is needed to investigate the relationship between kadsuralignan F treatment and the ubiquitination of tyrosinase.

## 3. Experimental Section

### 3.1. Reagents

Kojic acid, 3,4-dihydroxyphenilalanine (L-DOPA), tyrosine, mushroom tyrosinase, arbutin, cycloheximide, MG-132 and chloroquine were purchased from Sigma Chemical Co. (St. Louis, MO, USA). Protease inhibitor cocktail (CompleteTM) was from Roche Applied Science (Mannheim, Germany). Kadsuralignan F was isolated from *K. coccinea* and was purified by column chromatography, preparative TLC, and reverse-phase HPLC [21]. Briefly, dried *K. coccinea* (30 kg) was refluxed in 95% ethanol three times. All extracts were merged and then concentrated under vacuum to obtain a sticky solid (2.6 kg). The solid was suspended in water, and was then repeatedly extracted with petroleum ether, methylene chloride, ethyl acetate, and butanol. A residue extracted with methylene chloride (240 g) was separated by silica gel chromatography (gradient elution with benzene-acetic ether from 0:100 to 100:0) and was followed by HPLC. 12 fractions were gained from the extract, and the third fraction was concentrated and applied to silica gel chromatography and HPLC. Among them, kadsuralignan F (30.4 mg) was isolated. The structure of kadsuralignan F was elucidated by means of its physicochemical properties as determined through spectroscopic analyses.

### 3.2. Cell Culture

Melan-A cells were cultured in RPMI 1640 (Lonza ltd., Basel, Switzerland), supplemented with 10% heat-inactivated fetal bovine serum (Lonza ltd., Basel, Switzerland), 100 U/mL potassium penicillin and 100 mg/mL streptomycin sulfate (Lonza ltd., Basel, Switzerland), and phorbol 12-myristate 13-acetate (Sigma-Aldrich, St Louis, MO, USA). Cells were maintained with 10% CO_2_ in a humidified chamber (Thermoscientifics, Waltham, MA, USA) at 37 °C.

### 3.3. Measurement of Cell Viability

Cell viability was determined by using cell proliferation reagent WST-1 (Roche Applied Science, Mannheim, Germany) following the manufacturer’s Instructions. Briefly, 10 μL of WST-1 solution was added to each well containing melan-A cells which were cultured with kadsuralignan F for 72 h. After 4 h of incubation at 37 °C under 10% CO_2_, absorbance was measured using a SpectraMax 190 microplate reader (Molecular Devices Corp., Sunnyvale, CA, USA) at 450/690 nm. The percentage cytotoxicity was calculated by comparing data from treated cells with that of the control.

### 3.4. Determination of Melanin Contents

Melan-A cells were seeded into 48-well plate at 1.5 × 10^4^ cells/well. After 24 h of incubation, cells were treated with various concentrations of kadsuralignan F for 6 days. Every 3 days, media were removed and replaced with fresh media containing the samples. After treatment, the cells were washed with Dulbecco’s phosphate buffer saline (DPBS) and were dissolved in 2 N NaOH containing 10% DMSO for 1 h at 60 °C. Absorbance was measured at 475 nm, and melanin content was determined against a standard curve of synthetic melanin (Sigma-Aldrich, St. Louis, MO, USA). The values were normalized by the total protein contents in each sample.

### 3.5. Whitening Assessment in Human Skin Equivalents

Dark (from African-American skin) Human epidermal equivalents (MelanoDerm) were purchased from MatTek Corp. (Ashland, MA, USA). MelanoDerms were grown at the air–liquid interface, and the maintenance medium was replenished every 2 days. Kadsuralignan F was treated at 47.48 μM and 94.96 μM prepared in DPBS, which showed no cytotoxicity on skin equivalents. DPBS and kojic acid (1%) treatments were used for vehicle-treated and positive controls, respectively. Pigmentation of the skin equivalents was assessed by comparing the change in L* value, a value of CIE 1976 (L*, a*, b*) color space representing the brightness, as previously reported [31]. The level of pigmentation was monitored by calculating the difference (ΔL* value) between the mean L* values at day 9 and at day 0 for each skin equivalent.

### 3.6. Identification of Tyrosinase Inhibition Activities

For the mushroom tyrosinase assay, 0.1 M potassium phosphate buffer (pH 6.8) containing samples, mushroom tyrosinase (10 units), and tyrosine (0.55 mM) were incubated together at 37 °C for 10 min in a 96-well plate. After incubation, absorbance was measured by the microplate reader at a wavelength of 475 nm for tyrosine hydroxylation to DOPA. For cellular tyrosinase assay, total melan-A cell lysate was extracted by incubation in lysis buffer (0.1 M phosphate buffer, pH 6.8, 1% Triton X-100) at 4 °C for 1 h. The lysate was then centrifuged at 15,000× *g* for 30 min, and supernatant was collected. Cellular tyrosinase activity was assessed by reacting the mixture containing supernatant (40 μg), and L-DOPA (5.1 mM) with samples. During incubation at 37 °C for 30 min, absorbance was monitored at a wavelength of 475 nm in order to detect the conversion of DOPA to DOPA chrome via DOPA quinine.

### 3.7. Western Blot Analysis

Melan-A cells were cultured with samples for 72 h. Following harvesting and washing with DPBS, cells were either lysed in extraction buffer (0.1 M Tris-HCl, pH 7.2; 1% TritonX-100, 200 mM NaCl, protease inhibitor cocktail) at 4 °C. Each cell lysate (10 μg) was loaded onto 4~12% Bis-Tris sodium dodecyl sulfate/polyacrylamide gels for electrophoresis and was then transferred to nitrocellulose membranes (Invitrogen, Carlsbad, CA, USA). Membranes were blocked with 5% skim milk in Tris-buffered saline (TBS) containing 0.01% Tween-20 for 2 h at room temperature, before overnight incubation with primary antibody at 4 °C. The rabbit anti-tyrosinase, TRP-1 and TRP-2 antisera (αPEP7, αPEP1, and αPEP8, respectively) were a kind gift from Dr. V. J. Hearing (National Institutes of Health, Bethesda) and anti-microphthalmia-associated transcription factor (MITF) was purchased from Thermo Fisher Scientific (San Jose, CA, USA). After incubation, membranes were rinsed three times with TBS and were incubated with HRP-conjugated secondary antibodies (Santa Cruz, CA, USA) for 1 h at room temperature. After washing, membranes were subjected to Western Blotting Luminol Reagent (Santa Cruz, CA, USA) and were visualized using the LAS-3000 imaging system (Fuji Film, Tokyo, Japan).

### 3.8. RNA Isolation and Reverse Transcription Polymerase Chain Reaction (RT-PCR)

Melan-A cells cultured with samples for 24 h were washed twice with DPBS, and were lysed using Trizol (Invitrogen, Carlsbad, CA, USA) by vortexing and samples incubated for 10 min at room temperature followed by chloroform addition and centrifugation (12,000 rpm, 15 min, 4 °C). After centrifugation, the aqueous phase of the samples was collected and isopropanol was added. The mixture was incubated for 10 min at room temperature before centrifugation (12,000 rpm, 10 min, 4 °C). RNA pellets were washed with 75% ethanol and cleaned up using RNeasy mini kit (Qiagen, Inc., Valencia, CA, USA) according to the manufacturer’s instructions. RNA yield was estimated by determining the optical density at 260 nm. Subsequently, cDNA was synthesized from total RNA (4 μg) with reverse transcriptase (Superscript Reverse Transcriptase (RT) II kit, Invitrogen, Carlsbad, CA, USA) at 50 °C for 1 h, following a denaturing step at 95 °C for 5 min according to the manufacturer’s instructions. The cDNA was amplified in a reaction mixture containing TaqMan universal PCR master mix (Applied Biosystems, Foster city, CA, USA) and probes for TaqMan gene expression assay (Applied Biosystems, Foster city, CA, USA) by 7300 Real Time PCR System (Applied Biosystems, Foster city, CA, USA). Real-time quantitative PCR analysis was carried out under the following conditions: 40 cycles of denaturation at 95 °C for 15 s, annealing at 60 °C for 30 s, and a final extension at 72 °C for 60 s. Relative levels of each melanogenic protein mRNA was expressed compared to gapdh mRNA. The probes used were Mm00495817_m1 for tyrosinase, Mm00453201_m1 for tyrosinase related protein-1, Mm01225584_m1 for tyrosinase related protein-2, Mm00434954_m1 for mitf, and Mm99999915_g1 for gapdh.

### 3.9. Statistical Analysis

Statistical significance of all experimental data was determined by one-way ANOVA/Dunnett’s multiple comparison test, using MINITAB software program (14.0 for windows). Values of *p* < 0.05 were considered to be statistically significant.

## 4. Conclusions

In this study, we first reported that kadsuralignan F, a new dibenzocyclooctadiene lignan from a traditional medicine *K. coccinea*, showed whitening activity, as identified by the mechanism of kadsuralignan F on melanogenesis inhibition. We found that kadsuralignan F induced tyrosinase degradation via the proteasomal pathway, and subsequent melanin contents were reduced in melan-A cells and human skin equivalents. Overproduction and accumulation of melanin is related to hyperpigmentary skin disorders, and kadsuralignan F is an effective inhibitor of melanogenesis which can be useful as an effective skin-whitening agent.
